# Idiopathic Effusive-Constrictive Pericarditis Presented by Variant Angina

**DOI:** 10.7759/cureus.14380

**Published:** 2021-04-09

**Authors:** Kentaro Adachi, Takaharu Hayashi, Takashi Omatsu, Atsushi Hirayama, Yoshiharu Higuchi

**Affiliations:** 1 Cardiovascular Medicine, Osaka Police Hospital, Osaka, JPN

**Keywords:** effusive-constrictive pericarditis, variant angina, pericarditis, heart failure

## Abstract

An 80-year-old man presented to our hospital complaining of loss of appetite. During the medical examination, he developed variant angina accompanied with heart failure. Oral calcium channel blocker therapy controlled his variant angina, but medical management of heart failure became increasingly difficult due to gradually increasing pericardial effusion, and pericardiocentesis leading to the diagnosis of effusive-constrictive pericarditis (ECP). Here, we report a rare case of idiopathic pericarditis caused variant angina with already having endothelial dysfunction and eventually developed ECP.

## Introduction

The concept of effusive-constrictive pericarditis (ECP) was described many years ago by Burchell [1] and Spodick [2], but it was not well characterized. ECP is defined by concurrent pericardial effusion and pericardial constriction. The hallmark of ECP is the persistence of elevated right atrial pressure after intrapericardial pressure is normalized with the removal of pericardial fluid [3]. The underlying pathogenetic process predominantly involves the visceral pericardium and epicardium. It combines visceral pericardial constriction with pericardial inflammation. Variant angina was described by Prinzmetal and it consists of chest pain at rest, ST-elevation, and spontaneous resolution or resolution with the use of sublingual nitroglycerin [4]. The reported underlying mechanisms are autonomic nervous system, inflammation, endothelial dysfunction, smooth muscle cell hypercontractility, oxidative stress and genetics [5].

## Case presentation

**Figure 1:**
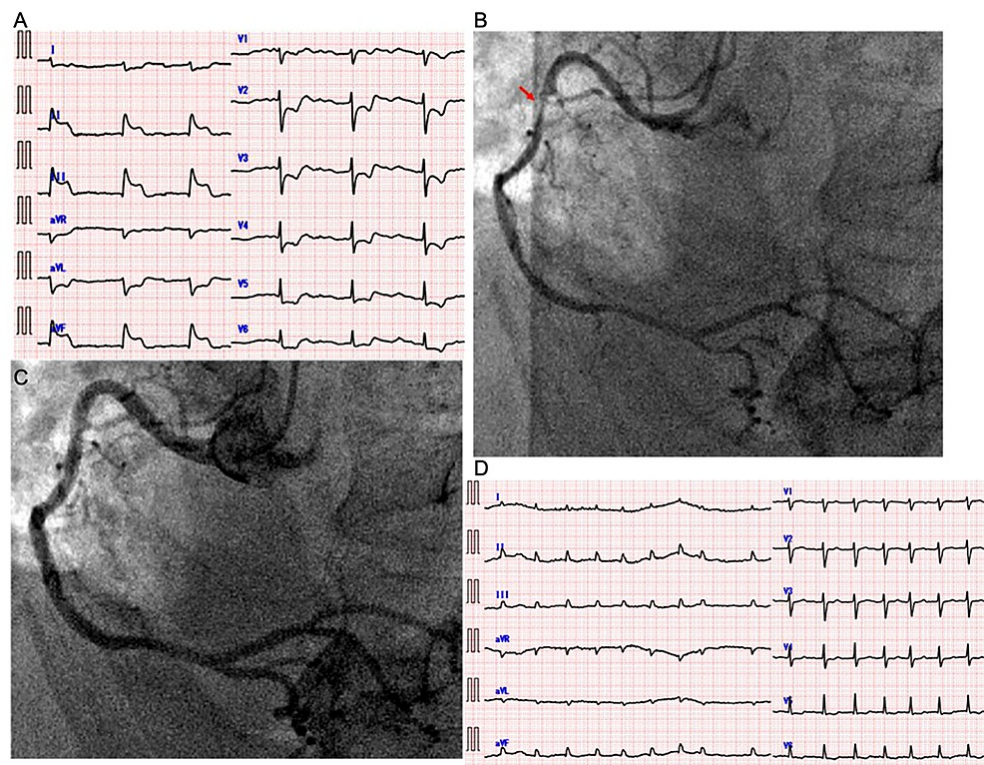
Variant angina A: Electrocardiography (ECG) showed remarkable ST elevation in the II, III and aVf leads and an escape rhythm. B: Prior to administration of nitroglycerin, there was significant stenosis in the proximal right coronary artery (red arrow). C: After administration of nitroglycerin, areas with coronary stenosis became sufficiently dilated. D: ECG in the catheterization laboratory showed resolution of ST elevation in the II, III and aVf leads and atrial fibrillation.

An 80-year-old man presented to our hospital complaining of abdominal fullness and loss of appetite that gradually worsened two weeks ago. He began to develop cold symptoms such as cough and sputum five weeks ago and became aware of dyspnea three weeks ago. He was seen and introduced to the department of gastroenterology for scrutiny by a home doctor. During the medical examination, he complained of chest pain and lost consciousness. Physical examination on admission revealed a heart rate of 56 beats/min and blood pressure of 87/65 mmHg. We could see remarkable coldness of limbs and systemic edema. At the age of 75, he received treatment for prostate cancer. His serum prostate-specific antigen (PSA) was high at 20.5 ng/ml. He did not receive chemotherapy or radiation therapy. At the age of 79, he was diagnosed with atrial fibrillation. His atrial fibrillation was followed up with anticoagulant therapy and heart rate control. He did not have any other cardiac, malignancy, inflammatory, exposure history. Electrocardiography (ECG) showed ST segments elevation in the II, III, aVf leads (Figure 1A).

Echocardiography revealed pericardial effusion and inferior asynergy. Laboratory findings on admission included a slight increase in markers of inflammation (Table 1).

**Table 1:** Laboratory findings on admission Laboratory tests showed high inflammatory markers and negative tumor markers. Interferon-gamma release assay for tuberculosis (T-spot) and ANA were both negative. First and fourth columns represent each inspection items, second and fifth columns are reference ranges, third and sixth columns are each item's values. WBC: White blood cells, RBC: Red blood cells, Hb: Hemoglobin, Hct: Hematocrit, CK: Creatine kinase, CK/MB: Creatine kinase myocardial band, AST: Aspartate aminotransferase, ALT: Alanine aminotransferase, LDH: Lactate dehydrogenase, BUN: Blood urea nitrogen, Cr: Creatinine, CRP: C-reactive protein, HbA1c: Hemoglobin A1c, UA: Uremic acid, T. Bil.: Total bilirubin, BNP: Brain natriuretic peptide, AFP: α-fetoprotein, CEA: Carcinoembryonic antigen, SCC: Squamous cell carcinoma, NSE: Neuron specific enolase, IGRA: Interferon gamma releasing assay, TSH: Thyroid stimulating hormone, BE: Base excess.

Laboratory data (on admission)					
WBC (/uL)	3500-9800	10000	AFP (ng/mL)	<10	2
Eosinophil (%)	0-10	0.2	CEA (ng/mL)	<5	2.3
RBC ( x 10^9^/uL)	4.3-5.7	4.7	SCC (ng/mL)	<1.5	0.7
Hb (g/dL)	13.5-17.6	14.8	SPAN-1 (U/mL)	<16.3	8
Ht (%)	39.8-51.8	44	NSE (ng/mL)	<30	14.1
Platelets ( x 10^4^/uL)	13.1-36.2	25.8	DUPAN-2 (U/mL)	<150	<25
CK (U/L)	30-200	94	IGRA		Negative
CK/MB (U/L)	<25	3	Anti-nuclear antibody		Negative
AST (U/L)	10-33	45	TSH (uU/mL)	0.3-4.9	8.4
ALT (U/L)	6-35	34	Free T4 (ng/mL)	0.7-1.5	1.1
LDH (U/L)	110-225	334	D-dimer (ug/mL)	<1	5.4
BUN (mg/dL)	8.4-20.4	35.5			
Cr (mg/dL)	0.6-1.0	1.47	pH	7.3-7.4	7.3
CRP (mg/dl)	<0.35	1.53	PaO2 (mmHg)	75-100	112.4
HbA1c		7.5	PaCO2 (mmHg)	35-45	30.7
UA (mg/dL)	2.2-6.7	9.1	HCO3^-^ (mmol/L)	21-29	16
T. Bil. (mg/dL)	0.2-1.2	1.8	BE (mmol/L)	-1.8-3.2	-8.6
BNP (pg/mL)	<18.4	101	Lactate (mg/mL)	4.5-18.0	33
Troponin T (ng/mL)	<0.1	0.01			

**Figure 2:**
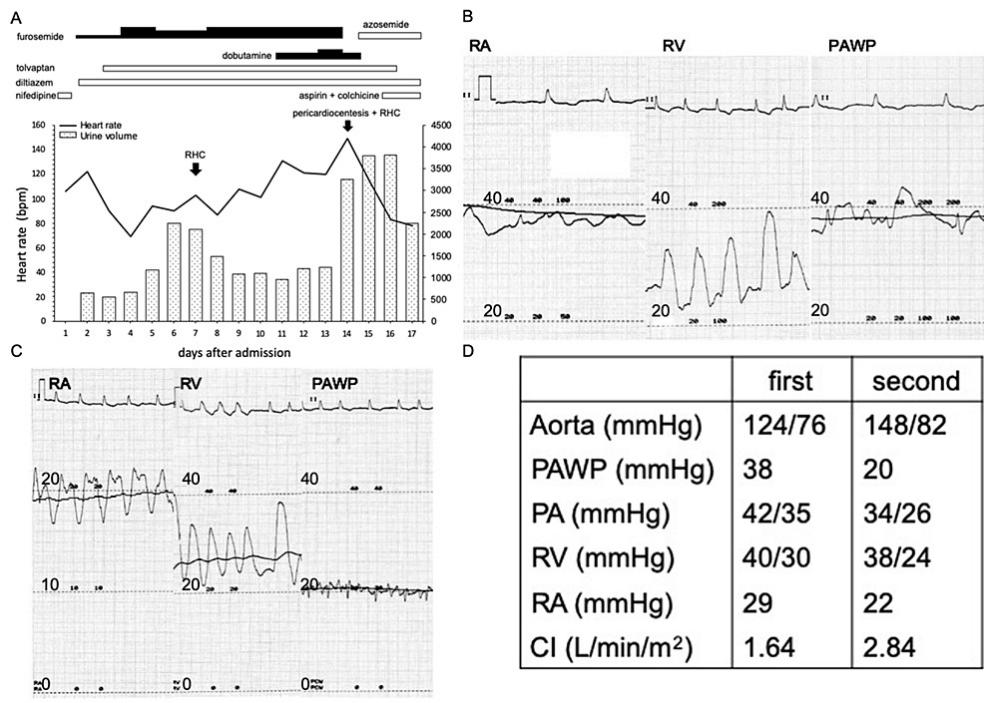
Clinical course during hospitalization and right heart catheterization performed before and after pericardiocentesis. A: Clinical course. The left vertical axis shows heart rate. The bar graph shows total daily urine volume in a day. Intravenous furosemide was started at 20 mg daily, which was increased to 80 mg daily. Dobutamine was administered starting at 2 mg/kg/min and increased to 3 mg/kg/min. Solid squares show intravenous medications. Open squares show oral medications. B: It showed RA, RV and PAWP pressure curves on hospital day 7. RA pressure and PAWP pressure were also high. C: Pressure curve tracings after pericardiocentesis. The range of RA pressure curves was different from the range of the other pressure curves. D: RHC values. After pericardiocentesis, cardiac index was improved, accompanied by decreases in PAWP and PA pressures. However, RA pressure remained high. CI: cardiac index, PA: pulmonary artery, PAWP: pulmonary artery wedge pressure, RA: right atrium, RHC: right heart catheterization, RV: right ventricular.

Enhanced computed tomography (CT) ruled out aortic dissection and calcium deposits in the pericardium. Thus, coronary angiography was performed (Figure 1B, 1C). It revealed neither occlusion nor significant stenosis after administration of nitroglycerin. ECG showed resolution of ST segments elevation (Figure 1D) by the time of coronary angiography. We diagnosed variant angina and started to administer a calcium channel blocker. His chest symptoms were well controlled but urine volume was remarkably decreased despite continuous intravenous administration of furosemide, body weight continuously increased with systemic edema. On hospital day 4, we started to administer tolvaptan (7.5 mg/day). After initiation of tolvaptan therapy, his urine volume was transiently increased (Figure 2A).

**Figure 3:**
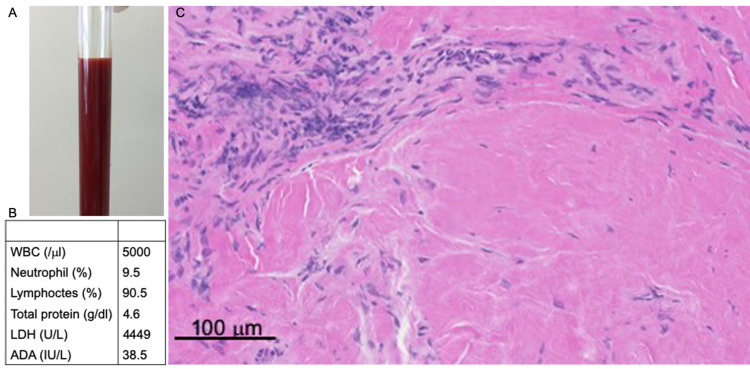
Pathological findings in the pericardium. A: The pericardial effusion fluid appeared bloody. B: There were many lymphocytes in this exudate. C: On pathologic examination, the specimen did not have any specific features. Lymphocytes had invaded in the pericardium, but there were no specific findings such as granuloma.

We performed right heart catheterization (RHC) because we suspected cardiac tamponade. RHC showed remarkably high right atrial pressure accompanied by pulmonary hypertension (Figure 2B) and low cardiac output. However, pericardiocentesis was challenging because of the location and amount of fluid. After RHC, his urine volume decreased each day. We added intravenous furosemide and dobutamine. On day 14, we performed pericardiocentesis for the pericardial effusion that had gradually enlarged and made difficult to maintain his hemodynamic status. We removed 350 mL of bloody pericardial fluid (Figure 3A, 3B).

After pericardiocentesis, RHC showed improved cardiac output but right atrial pressure remained high (Figure 2C, 2D). Echocardiography showed that the posterior wall was flattened at the end of left ventricular diastole and the ventricular septum bounced toward the left ventricle during inspiration. After pericardial drainage, the pericardium remained hardened. Based on these findings, we diagnosed ECP. Immediately after pericardiocentesis, his urine volume increased and heart rate decreased. Polymerase chain reaction and culture of the pericardial fluid yielded negative culture. Cytological examination indicated no malignant cells. Epicardial biopsy eventually led to the diagnosis of idiopathic ECP because there are no specific findings (Figure 3C). We prescribed colchicine (0.5 mg/day) and high-dose of aspirin (1,000 mg/day). Recurrence of pericardial effusion has not been observed during two years of follow-up.

## Discussion

The causes and the mechanisms underlying the development of variant angina are still poorly defined and are likely multifactorial. Autonomic nervous system, inflammation, endothelial dysfunction, smooth muscle cell hypercontractility, oxidative stress and genetics were proposed mechanisms of variant angina. Especially, endothelial dysfunction is one of the most important factors for the induction of variant angina [5]. Coronary artery spasm happens due to vasoactive substances such as thromboxane A2 (TxA2) [6], which are released during inflammation caused coronary endothelial dysfunction. Pericarditis is also an inflammatory disease and high concentration of TxA2 is produced. The risk factors of endothelial dysfunction like diabetes mellitus, marijuana, hypertension, age, inflammation and smoking have been established [7]. Recently, endothelial dysfunction due to atrial fibrillation has been proposed [8]. Turbulent coronary blood flow due to atrial fibrillation increases shear stress in blood vessels, resulting in endothelial dysfunction. Virchow’s triad suggests that endothelial injury occurs in patients with cancer. PSA has been identified as a member of the human kallikrein family of serine proteases [9]. Numerous observations suggest that the activity of the kallikrein-kinin system, which has also bradykinin-generating activity, is related to inflammation and endothelial dysfunction and can lead to cardiovascular diseases [10]. In this case, variant angina was induced by pericarditis in a patient with endothelial dysfunction due to prostate cancer and atrial fibrillation.

ECP developed due to a gradually enlarging pericardial effusion generated by pericarditis. We added inotropic agents and large amount of intravenous furosemide to treat congestion, but gradually his renal function worsened. Intravenous loop diuretics are mainly used for the purpose of decongestion [11]. Their use has been shown to be associated with an increased risk of worsening renal function [12]. Worsening renal function during hospitalization for acute decompensated heart failure is a powerful prognostic factor [13]. We hesitated to add more loop diuretics for decongestion, because of concern about further exacerbation through cardiorenal syndrome due to deterioration of renal function. Tolvaptan, a selective V2 receptor antagonist, can alleviate congestion with a significantly lower risk of worsening renal function in acute decompensated heart failure [14]. One Japanese group showed that tolvaptan was remarkably effective for inoperative constrictive pericarditis [15]. We administered tolvaptan for the purpose of decongestion, but its effect was just transient and finally we performed pericardiocentesis, leading to diagnosis of ECP. Diuretics must be applied cautiously as there is already decreased cardiac output. In this case, pericardiocentesis was required to relieve cardiac compression.

Symptomatically, affected patients may be treated with nonsteroidal anti-inflammatory drugs (NSAIDs), colchicine, or steroids for the pain and inflammation. Colchicine has a known anti-inflammatory effect blocking tubulin polymerization with consequent impaired microtubule assembly, thus inhibiting inflammasome formation and cytokine release [16]. Treatment of the underlying disorder is also necessary for decreasing the inflammatory cycle. It is important to suppress pericardial inflammation because continuous inflammation would not only produce pericardial effusion but also cause recurrence. Long-term prognosis in affected patients remains good, and pericardiectomy is rarely required [17]. In the case of recurrence, pericardiectomy should be considered. Several causes of ECP are recognized, such as cardiac surgery, infection (especially tuberculosis), radiation, collagen disease, trauma and malignancy [3]. However, approximately half of ECP cases are considered idiopathic. Based on several clinical examinations, we diagnosed idiopathic ECP in this patient. We concluded that pericarditis triggered variant angina and ECP in this patient, who had a thickened and hardened pericardium.

## Conclusions

Although rare, variant angina may become apparent with pericarditis in the context of endothelial dysfunction. In our case, endothelial dysfunction was caused by atrial fibrillation and prostate cancer and pericarditis was a trigger of variant angina through TxA2. A pericardial effusion produced by pericarditis and increasing over time eventually caused ECP. An effusive-constrictive pericarditis is often difficult to diagnose, but we need to keep in mind the possibilities if it is hard to manage accompanied by pericardial effusion.
